# Incorporation of *Gene Function and Disease* Into *CFG*

**DOI:** 10.1002/cfg.299

**Published:** 2003-06

**Authors:** Steve Oliver


					Comparative and Functional Genomics
Comp Funct Genom 2003; 4: 286.
Published online in Wiley InterScience (www.interscience.wiley.com). DOI: 10.1002/cfg.299
Editorial
Incorporation of Gene Function and Disease into CFG
With this issue, our sister journal, Gene Function
& Disease (GFD), published by Wiley-VCH, will
cease to be a separate title and will be incorporated
into Comparative and Functional Genomics.
In combining the contents from these two jour-
nals, CFG aims to provide a more comprehen-
sive coverage of genomic research, including more
studies applying global approaches to the genet-
ics of human disease. In particular, CFG would
like to encourage the submission of papers and
reviews covering protein structure prediction and
modelling, in relation to understanding protein
function and interactions. With this in mind, we
are delighted to welcome Thomas Lengauer (Max-
Planck-Institut fur Informatik, Germany) onto our
Editorial Board.
Professor Walter Doerfler and Petra Bohm, with
the support of their Editorial Board, did a great
job launching GFD. The merger of these two
journals will ensure that the foresight and expertise
that they brought to GFD will go on to enrich
CFG, benefiting the authors and readers of both
journals.
Steve Oliver
Editor-in-Chief
Copyright  2003 John Wiley & Sons, Ltd.

				

